# Isolation of a multi-trait plant growth promoting *Brevundimonas* sp. and its effect on the growth of *Bt*-cotton

**DOI:** 10.1007/s13205-013-0126-4

**Published:** 2013-03-13

**Authors:** Varun Kumar, Rajesh Gera

**Affiliations:** 1 Department of Microbiology, CCS Haryana Agricultural University, Hisar, 125004 India; 2 Department of Biotechnology and Bioinformatics, Jaypee University of Information Technology, Waknaghat, 173234 HP India

**Keywords:** Plant growth promoting rhizobacteria, *nif*H, Indole-3-acetic acid, *Bt*-cotton, *Brevundimonas* sp.

## Abstract

Arid regions pose a serious problem for crop production by suppressing plant growth. The use of plant growth promoting rhizobacteria (PGPR) as bioinoculant may be promising to enhance the crop yield in arid conditions. In the present investigation, four strains of cultivable bacteria associated with the rhizosphere of *Saccharum* L. grown in arid region were isolated using N-free media. Assessment of their nitrogen-fixing ability through amplification of *nif*H gene showed the presence of *nif*H gene (390 bp) in only one (MDB4) of the four isolates. The nitrogen-fixing potential of this isolate was confirmed by the presence of nitrogenase activity determined using acetylene reduction assay. The diazotrophic MDB4 isolate also exhibited other PGPR traits, such as the production of indole-3-acetic acid (IAA) and ammonia. In pot experiments, inoculation of *Bt*-cotton seeds with MDB4 enhanced the growth of plants as shown by significant increase in plant height (68.41 %), shoot dry weight (58.44 %) and root dry weight (64.81 %) over untreated control. The MDB4 strain was Gram negative and identified as *Brevundimonas* sp. on the basis of phenotypic, biochemical, phylogenetic and 16S rRNA gene sequencing data. It is concluded that the MDB4 bacterial strain having different plant growth promoting activities can be considered as a beneficial microbe for sustainable agriculture in arid regions.

## Introduction

The arid zone of India covers an area of 38.7 million hectare (m ha), out of which 31.7 m ha comes under hot arid zone (Gothwal et al. 2008). In the arid zone, temperature raises up to 50 °C with average rainfall <200 mm causing severe conditions for most of the life forms besides poor nutritional status of such soils (Bhatnagar and Bhatnagar 2005). The soil fertility in these areas can be enhanced using bioinoculants. The use of plant growth-promoting rhizobacteria (PGPR) is also known to improve plant growth and yield (Vessey 2003; Abbasi et al. 2011).

PGPR are native soil bacteria that colonize the rhizosphere or plant roots resulting in stimulation of plant growth either directly and/or indirectly. In the recent years, PGPR have received worldwide importance for agricultural benefits, as a diverse array of bacteria, including species of *Rhizobium*, *Agrobacterium, Pseudomonas*, *Azospirillum*, *Azotobacter*, *Bacillus, Klebsiella* and many others that have been shown to facilitate plant growth both under greenhouse and field conditions (Farina et al. 2012). The use of PGPR is steadily increasing in agriculture and offers an attractive way to replace chemical fertilizers, pesticides and supplements (Rani and Arundhati 2012). The mechanisms through which PGPR stimulate plant growth and development are not fully understood, but are believed to include (a) production of phytohormones; (b) nitrogen fixation; (c) solubilization of inorganic phosphate and mineralization of organic phosphate; (d) production of siderophores that chelate iron and make it available to plant root; and (e) antagonism against phytopathogenic microorganisms by the synthesis of antibiotics, enzymes or fungicidal compounds, as well as competition with harmful microorganisms (Glick 1995; Weyens et al. 2009; Abbasi et al. 2011).

Biological nitrogen fixation by bacteria present in the rhizosphere is an important property contributing to plant growth (Gothwal et al. 2008). Nitrogenase complex, the enzyme that catalyzes biological dinitrogen reduction to ammonium, is composed of two highly conserved proteins: the iron protein, also known as nitrogenise reductase (encoded by the *nifH* gene) and the molybdenum iron protein, also known as dinitrogenase (encoded by the *nifDK* genes). The *nif*H genes encoding the iron-protein component of nitrogenase enzyme are evolutionarily conserved and highly valuable for phylogenetic analysis and detection and identification of diazotrophs by cultivation-independent methods (Ueda et al. 1995).

Although *Brevundimonas* sp. has been used as PGPR in enhancing growth of wheat plants (Rana et al. 2011), its effect on the growth of *Bt*-cotton has not yet been investigated. Cotton is an important commercial crop which meets nearly 75 % of total raw material needs of the textile industry in India. Research on cotton under various soil and climatic conditions has revealed the beneficial effect of nitrogen application on growth, yield and quality of cotton (Narayanan et al. 1974). Thus, it was envisaged that inoculation of cotton with diazotrophic PGPR would enhance the plant growth by supplying nitrogen. The present study reports the isolation and identification of multi-trait *Brevundimonas* sp. having nitrogen fixing ability as well as other plant growth promoting activities and evaluation of its effect on the growth of *Bt*-cotton. This is the first report on *Brevundimonas* sp. from arid region that elicits plant growth promotion on *Bt*-cotton plant.

## Materials and methods

A rhizospheric soil sample of sugarcane (*Saccharum* L.) collected from Bhoria khera, district Sirsa (latitude: 29°30′N, longitude: 75°18′ E), an arid region of Haryana (India), was used for isolation of PGPR. Bacteria from the soil sample were isolated by serial dilution method on DB agar plates which contained (in g/L); malic acid (5.0), dipotassium hydrogen phosphate (0.6), potassium dihydrogen orthophosphate (0.4), manganese sulphate (0.01), magnesium sulphate (0.05), sodium chloride (0.02), sodium molybdate (0.002), potassium hydroxide (4.0) and bromothymol blue (2 mL of 0.5 % alcoholic solution), pH 6.8. The plates were incubated at 37 °C in a bacteriological incubator. After 4–5 days of incubation, distinct morphotypes of bacteria were screened on the basis of colony color, shape and size. Each morphotype was purified by re-streaking on fresh DB agar plate and the purified isolates were maintained on DB slants at 4 °C.

Genomic DNA of each morphotype was isolated using CTAB method (Ausubel et al. 1987) and subjected to the amplification of *nif*H gene using *nif*H primers; 19F (5′-GCIWTYTAYGGIAARGGIGG-3′) and 407R (5′-AAICCRCCRCAIACIACRTC-3′) (Ueda et al. 1995). The PCR reaction mixture (25 μl) contained 2 μl template DNA (0.1–0.14 μg μl^−1^), 2 μl of each primer (10 pmol each), 12.5 μl of Red Taq Ready Mix (Bangalore Genei, India) and 6.5 μl sterile water. The conditions for PCR were: initial denaturation at 95 °C for 4 min, 35 cycles of denaturation at 94 °C for 30 s each, annealing at 54 °C for 1 min and extension at 72 °C for 1 min followed by a final extension at 72 °C for 5 min. The PCR was then set on hold at 4 °C. The amplified *nif*H gene was checked by 2 % agarose gel electrophoresis and visualized by observing UV fluorescence using gel documentation system (MiniBis Pro, DNR Bio Imaging Systems, US).

The nitrogen-fixing potential of the bacterial isolates was evaluated by measuring their nitrogenase activity using acetylene reduction assay (Boddey 1987). Plant growth promoting abilities were determined as per the standard methods: IAA (Gordon and Weber 1951), phosphate solubilization (Pikovskaya 1948), ammonia production (Cappuccino and Sherman 1992) and siderophore production (Schwyn and Neilands 1987).

The effect of the PGPR isolate on the growth of *Bt*-cotton plants was determined through a pot culture experiment. The experiment was conducted in a completely randomized design, with three replicates. The *Bt*-cotton crop was raised in earthen pots (8″ size) filled with 5-kg non-sterile soil taken from CCS HAU fields (latitude: 29°10′N and longitude: 75°46′E) and irrigated regularly to maintain 60 % water holding capacity of the soil. The recommended doses of fertilizers NPK (urea, single super phosphate and Muriate of potash in the ratio 70:27:10 kg^−1^ ha^−1^) were applied before sowing. Seeds of *Bt*-cotton (var. RCH 134) were surface sterilized by exposing these to 95 % ethanol followed by immersing in 0.2 % mercuric chloride solution for 3 min and rinsing ten times with sterile distilled water. The bacterial culture was grown in LB medium at 28 °C to attain 0.6 O.D. at 540 nm (10^8^–10^9^ cells mL^−1^). 1 mL of this overnight grown bacterial suspension was applied on each *Bt*-cotton seed for 10 min followed by sowing of the seeds (5 in each pot) to a depth of 5 mm. In control, seeds were treated with sterile LB medium instead of the bacterial suspension. The seeds of *Bt*-cotton (var. RCH 134) were also inoculated with the reference bacterial strain (HT-54) in a manner similar to MDB4 for comparison. The pots were kept in sunlight. After emergence, seedlings were thinned to three per pot. Plants were irrigated with only distilled water (without micro and macro nutrients). Plants were harvested after 90 days of sowing and dried to a constant weight at 65 °C. Plant height and dry weight of the plants were determined. Statistical analysis was performed by subjecting the triplicate sets of data to ANOVA (Analysis of Variance) using MINITAB version 15.0 and Tukey’s tests (*P* = 0.05).

Biochemical characteristics of the MDB strain were determined using HiCarbohydrate™ Kit (HiMedia, India). Oxidase test was performed by dispensing a bacterial colony on the filter paper soaked in*N*,*N*,*N*′,*N*′-tetramethyl-*p*-phenylenediamine dihydrochloride and examining it for the appearance of blue color which indicates a positive test. Catalase activity was estimated by monitoring the production of bubbles from 3 % hydrogen peroxide solution. Gram staining of the bacterial isolate was performed as per the method of Beveridge (2001). PCR amplification of 16S rRNA gene was carried out using primers fD1 (5′-AGA GTT TGA TCC TGG CTC AG-3′) and rP2 (5′-ACG GCT ACC TTG TTA CGA CTT-3′) (Weisburg et al. 1991). PCR reaction mixture (50 μl) contained 2 μl template DNA (50–70 ng μl^−1^), 2 μl of each primer (10 μM), 1 μl of 10 mM dNTPs, 5 μl of Taq buffer (10X), 1 μl of Taq DNA polymerase (3U μl^−1^) (Fermentas, US) and 12.0 μl of Millipore water. The conditions used for PCR were: initial denaturation at 94 °C for 3 min, 40 cycles of denaturation at 94 °C for 30 s each, annealing at 50 °C for 30 s and extension at 72 °C for 1 min followed by a final extension at 72 °C for 10 min. The PCR was then set on hold at 4 °C. PCR products were analysed by 1.5 % agarose gel electrophoresis and visualized by observing UV fluorescence using gel documentation system. The purified 16S rRNA gene was sequenced (Bioserve, Hyderabad, India) by primer walking using five different internal primers (16SEQ2R, 16SEQ3F, INS16SREV, 16SEQ4R and 16SEQ4F) and the sequence obtained in FASTA format was compared to the GenBank database using the algorithm BLASTN program to identify the most similar 16S rRNA gene sequences. The reference sequences were downloaded in FASTA format from NCBI database (http://www.ncbi.nlm.nih.gov) and the phylogenetic tree was constructed by the neighbour-joining method (Saitou and Nei 1987) using MEGA4 software (Tamura et al. 2007). Tree topologies were evaluated by performing bootstrap analyses using 1,000 re-samplings.

## Results and discussion

Plant growth promoting rhizobacteria enhance growth and development of plants by exploiting different mechanisms. In this study, we have isolated a multi-trait PGPR in nitrogen-free media from the rhizosphere of *Saccharum* L. grown in the arid region and used it as bioinoculant for *Bt*-cotton. On pouring the serially diluted soil sample on DB agar plates, four distinct bacterial morphotypes (MDB1, MDB2, MDB3 and MDB4) were obtained on the basis of their colony color, shape and size. To test the nitrogen fixing ability of the isolates, genomic DNA from all the four strains was isolated and subjected to amplification of the *nif*H gene. The results revealed that only one (MDB4) of the four strains showed *nif*H gene amplification with size of the amplified product as 390 bp. Attempts to amplify *nif*H from genomic DNA of the remaining three isolates using different sets of primers (Sarita et al. 2008) also failed indicating the absence of nitrogen fixing genes in these strains. Since the *nif*H gene is evolutionarily conserved among diazotrophs and could be used for their identification (Ueda et al. 1995), the presence of the *nif*H gene indicated that the MDB4 isolate was capable of nitrogen fixation. The MDB4 strain exhibited nitrogenase activity (47.90 nmol ethylene mg^−1^ protein h^−1^) which confirmed its nitrogen-fixing ability.

The evaluation of other plant growth promoting activities of the strain MDB4 revealed that it produced indole-3-acetic acid (364.1 ± 0.750 μgmL^−1^) and ammonia (0.954 ± 0.006 μgmL^−1^); however, it neither solubilized phosphorus in Pikovskaya medium containing tricalcium phosphate nor produced siderophores. Thus, the bacterial strain isolated from the sugarcane rhizoshere was a multi-trait PGPR which exhibited nitrogen-fixing ability and production of indole-3-acetic acid as well as ammonia. These plant growth promoting characteristics of the isolated strain would enhance the growth and yield of plants as suggested by several researchers (Farina et al. 2012; Glick 1995). The production of phytohormones is usually used to explain the various direct effects of PGPR on plants (Patten and Glick 1996). PGPR with the ability to fix biological nitrogen would have an advantage in counterbalancing the loss of nitrogen from soils.

The potential of the multi-trait MDB4 isolate on plant growth promotion was evaluated by using it as bioinoculant for *Bt*-cotton in pot experiments. A reference strain (HT-54) was also used as inoculant for comparison. Data obtained from this experiment showed stimulatory effects on plant height and dry weights of root and shoot after inoculation with MDB4 isolate and HT-54. The increase in plant height, shoot dry weight and root dry weight on inoculation with MDB4 strain was 68.41, 58.44 and 64.81 %, respectively, whereas the corresponding increase on inoculation with HT-54 strain was 48.96, 10.23 and 5.55 % (Table 1). So, the MDB4 strain was found to be more effective as compared to HT-54 in promoting plant growth which could be attributed to its nitrogen-fixing ability. A similar increase in biomass of canola plants following inoculation with bacterial strains *Achromobacter*, *Klebsiella*, *Pseudomonas*, *Pantoea* and *Chryseobacterium* has been reported (Farina et al. 2012). Nitrogen is one of the factors that directly influence vegetative growth and dry matter production. Decreased dry matter production associated with boll shedding process in cotton was due to nitrogen deficiency (Jackson and Gerik 1990). Ian (2006) indicated that nitrogen enhanced the *cry* protein concentration in the cotton plant which gave more protection to the crop from pest incidence. These reports might imply that diazotrophic nature of MDB4 was one of the factors responsible for enhanced plant height and dry weight. These findings implied that the isolated PGPR strain could be considered as plant growth promoter.

**Table 1:** Plant height (H), Shoot dry weight (S) and Root dry weight (R) of *Bt* cotton plant inoculated with HT-54 (reference strain) and MDB4 isolate

Treatment	Plant height (cm)	Shoot dry weight (g/plant)	Root dry weight (g/plant)	Difference from control		
				H	S	R
Control	29.98 ± 0.60	6.45 ± 0.38	0.91 ± 0.08	–	–	–
HT-54	44.66 ± 1.88	7.11 ± 0.39	1.14 ± 0.07	14.68***	0.66^#^	0.23^#^
MDB4	50.49 ± 1.81	10.22 ± 0.69	1.89 ± 0.24	20.51***	3.77**	0.98*

Group 1: Plant height; Group 2: Shoot dry weight; Group 3: Root dry weight. Data represent the mean ± SEM in each group. Comparisons were also made between group 1 and group 2 (*P* < 0.001); group 1 and group 3 (*P* < 0.001); group 2 and 3 (*P* < 0.05)

*** *P* < 0.001; ** *P* < 0.01; * *P* < 0.05 and ^#^non-significant

**Fig. 1:**
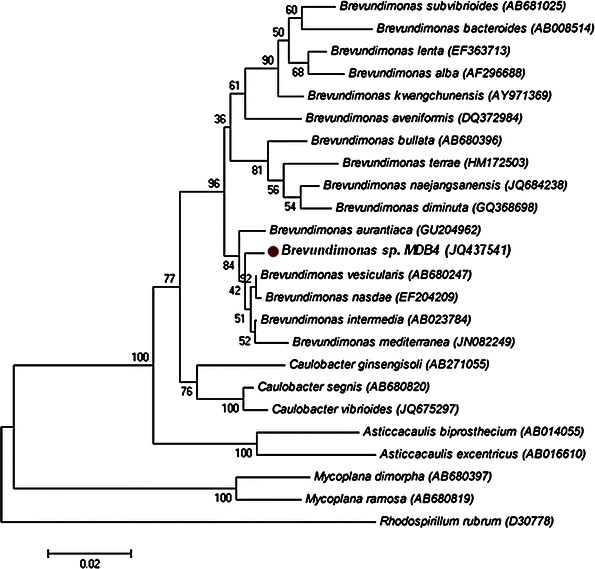
Phylogenetic tree showing the position of strain MDB4, *Brevundimonas* species and some other related taxa (Genbank accession numbers are given in parentheses) based on 16S rDNA gene sequences. Distances were calculated by neighbour-joining method. Numbers at branch points are bootstrap values (expressed as percentages of 1,000 replications). *Rhodospirillum rubrum* D30778 was used as an outgroup. *Bar* 0.02 substitutions per nucleotide position

The evaluation of biochemical characteristics of the strain MDB4 showed it positive for catalase and oxidase, but negative for urease production, starch hydrolysis and nitrate reduction. The strain was also able to utilize carbohydrates viz. d-glucose, galactose, maltose and cellobiose as energy sources. Mannose, trehalose, sucrose, mannitol, fructose, ribose, xylose and raffinose were not utilized as energy sources. These tests helped in identification of its genera as *Brevundimonas* (Holt et al. 1994). The partial 16S rRNA gene sequence of multi-trait PGPR strain obtained in this study was submitted in GenBank database (http://www.ncbi.nlm.nih.gov) under the accession number JQ437541 (*Brevundimonas* sp. MDB4). The 16S rRNA gene sequencing data of the bacterial isolate MDB4 showed 99 % similarity with the *Brevundimonas* sp. In the phylogenetic tree based on the neighbour joining method, the strain MDB4 fell within the cluster comprising *Brevundimonas* species (Fig. 1). *Brevundimonas* sp. MDB4 exhibited 16S rRNA gene sequence similarity values of 88.7–99.5 % to the type strains of *Brevundimonas* species and 82.2–99.2 % to other species included in the phylogenetic analysis. Identification of *Brevundimonas* sp. using 16S rRNA gene sequencing was supported by many researchers (Yoon et al. 2007). On the basis of phenotypic, phylogenetic and 16S rRNA gene sequencing data, the MDB4 isolate was identified as *Brevundimonas* sp.

Overall, the plant growth promoting properties of the *Brevundimonas* sp. MDB4 and its effect on *Bt*-cotton plant growth under control conditions suggest that this strain has a potential of being developed as bio-inoculant and is likely to be a promising strain for application in agriculture under Arid regions.
